# High dietary cation and anion difference formulation increased heat dissipation in non-lactating goats fed at high ambient temperature

**DOI:** 10.14202/vetworld.2023.2403-2410

**Published:** 2023-12-05

**Authors:** Thiet Nguyen, Narongsak Chaiyabutr, Sapon Semsirmboon, Somchai Chanpongsang, Sumpun Thammacharoen

**Affiliations:** 1 Department of Agricultural Technology, College of Rural Development, Can Tho University, Vietnam; 2 Department of Physiology, Faculty of Veterinary Science, Chulalongkorn University, Bangkok, 10330, Thailand; 3 The Academy of Science, The Royal Society of Thailand, Dusit, Bangkok 10300, Thailand; 4 Department of Husbandry, Faculty of Veterinary Science, Chulalongkorn University, Bangkok, 10330, Thailand.

**Keywords:** dietary cation-anion difference, goats, meal pattern, physiological responses, water balance

## Abstract

**Background and Aim:**

In our previous study, we observed that a high dietary cation and anion difference (DCAD) of 40 mEq/kg dry matter (DM) in the diets of lactating dairy goats increased heat dissipation. In the present study, we believe that the level of DCAD fed to non-lactating and non-pregnant goats was twice as high as that fed to lactating goats in our previous study. This increase could have resulted in a greater water balance due to increased intake of water and unchanged urinary excretion. Therefore, this study aimed to determine the behavioral and heat dissipation effects of a dietary shift from low to high DCAD levels in dairy goats under tropical conditions.

**Materials and Methods:**

Seven non-lactating and non-pregnant crossbred goats were used in this study. All animals were initially fed a low DCAD (15 mEq/100 g DM) diet from days 0–6 and then switched to a high DCAD (89 mEq/100 g DM) diet from day 7 (high DCAD-7) to day 18 (high DCAD-18).

**Results:**

The results revealed that a high DCAD increased DM intake from days 13–18 (p < 0.05). The larger daily meal size associated with the high DCAD-18 group was due to increased daytime meal sizes, not nighttime when compared to the low DCAD group. Dietary cation and anion difference supplementation did not affect daily water intake; however, drinking patterns differed between the low DCAD group and the high DCAD-7 group from 07:00 to 09:00 and during nighttime. Similarly, daily urine volume was unaffected by DCAD supplementation, but urinary patterns differed between the low DCAD and high DCAD-18 groups. The daily water balance remained unchanged across all treatments, yet, a higher morning water balance was observed in the high DCAD group. The high DCAD diet led to an increase in respiration rate and rectal temperature compared to the low DCAD diet.

**Conclusion:**

The observed eating, drinking, and urinary patterns collectively suggested that high DCAD supplementation mitigates the effects of heat stress in non-lactating goats fed at high ambient temperatures.

## Introduction

In tropical regions, low productivity is a major challenge for cattle and goats. Various factors, such as environmental conditions, nutrition, disease, and reduced genetic potential, can influence the productivity of animals in tropical countries. Notably, dairy animals raised in Thailand are often adversely affected by high ambient temperature (HTa) conditions. These conditions could be partially characterized by physiological changes that impact animal behaviors, including alterations in body temperature, respiratory rate, eating and drinking patterns, and urinary excretion. The previous studies by Salama *et al*. [1], Saipin *et al*. [2], and Thammacharoen *et al*. [3] have reported decreased dry matter intake (DMI) and milk yield in dairy animals under HTa conditions. In addition, low DMI induces a decrease in essential mineral intake under HTa conditions. Consequently, supplementing minerals in the diet has become a viable strategy to meet the mineral element requirements in high-temperature environments [4].

The previous studies by West *et al*. [5], Delaquis and Block [6], and Khelil-Arfa *et al*. [7] have reported that dairy cows supplemented with high levels of dietary cation and anion difference (DCAD) exhibited increased DMI, water intake (WI), and urinary excretion. Similarly, our previous studies on lactating goats have demonstrated the effect of high DCAD on eating and drinking behaviors, which subsequently influenced urine output and water balance [8,9]. We observed that high DCAD tends to increase heat dissipation in lactating dairy goats. The rate of increase in rectal temperature (Tr) was found to be lower in the high DCAD regimen [10]. However, our previous findings might be partially influenced by the high metabolic rate during lactation and the high DCAD of 40 mEq/kg DM in the diets. In addition, we believe that the level of DCAD fed to non-lactating and non-pregnant goats in this study was twice that of our previous study on lactating goats. This difference may lead to a greater water balance through increased water intake and unchanged urinary excretion. We hypothesize that by maintaining lower metabolic demand in non-lactating and non-pregnant goats, the heat dissipation effect of a high DCAD regimen can be clearly demonstrated through the relationship between core temperature and breathing.

Therefore, we aimed to determine the behavioral and heat dissipation effects of a dietary shift from a low to high DCAD regimen in dairy goats under tropical conditions.

## Materials and Methods

### Ethical approval

The procedures of this experiment were approved by Animals Care and Use Committee, Faculty of Veterinary Science, Chulalongkorn University (#1531074).

### Study period and location

The study was conducted during the summer season (April) 2020 at Nakornpathom training farm, Nakornpathom province, Thailand.

### Animals and management

Seven non-lactating and non-pregnant crossbred Saanen goats, with an average body weight of 27.08 ± 2.37 kg, were used in this experiment. These animals were individually housed in metabolic cages within two m-shaped pens with plastic floors for a 7-day adaptation period. Subsequently, the goats were fed low DCAD (15 mEq/kg DM) diets from days 0 to 6 and then switched to high DCAD (89 mEq/kg DM) diets from day 7 until the end of the experiment (day 18). The mineral composition of the diets is presented in Table-1. The DCAD levels in the diets were adjusted by adding NaHCO_3_ and K_2_CO_3_ (Table-1). The experimental diets, containing 44% corn silage and 56% concentrate (Table-1), were mixed into a total mixed ration (TMR). The corn silage used in the diets, derived from corn stover and approximately 1-1.5 meters in length, was chopped to a length of 5 cm. The goats were provided with TMR (refusals always exceeded 10% of the experimental diets) twice daily (at 07:00 and 14:00) and free access to water. The average temperatures, relative humidity, and temperature-humidity index (THI) recorded during the experiment at 07.00, 09.00, 11.00, 13.00, 15.00, 17.00, and 19.00 are presented in Table-2.

**Table-1:** Ingredients and chemical composition of experimental diets.

Diet composition	Low DCAD	High DCAD
Ingredients, g/kg DM		
Corn silage	440	440
Corn meal	257.8	237.6
Soybean meal	196.2	193.6
Cassava	32.6	15.0
Molasses	36.9	27.7
Rice bran	22.5	22.5
Premix	5.0	5.0
Limestone	9.0	9.0
NaHCO_3_	0	12.0
K_2_CO_3_	0	38.0
Chemical composition, g/kg DM		
DM	351.6	351.2
CP	167.3	167.2
NEL, Mcal/kg DM	1.66	1.67
Ca	5.2	5.4
P	3.6	4.9
Na	1.6	4.8
K	12.3	34.6
Cl	3.7	2.5
S	1.9	1.9
DCAD, mEq/100 g DM	15.59	89.05

NEL calculated according to NRC (1981); Low DCAD=Na+K – Cl – S: 15.59 mEq/100 g DM; High DCAD=Na+K – Cl – S: 89.05 mEq/100 g DM. DCAD=Dietary cation and anion difference, DM=Dry matter

**Table-2:** The averaged ambient conditions during daytime from the present experiment.

Item	07:00 h	09:00 h	11:00 h	13:00 h	15:00 h	17:00 h	19:00 h
Ta							
Low DCAD	26.50 ± 0.50	28.50 ± 0.50	29.50 ± 0.50	32.00 ± 1.00	33.00 ± 1.00	31.00 ± 1.00	27.50 ± 1.50
High DCAD-7	26.00 ± 1.00	28.50 ± 0.50	31.00 ± 1.00	32.50 ± 0.50	33.50 ± 0.50	32.50 ± 0.50	27.00 ± 0.50
High DCAD-18	26.00 ± 1.00	29.00 ± 1.00	31.50 ± 0.50	34.00 ± 0.50	34.00 ± 0.50	32.50 ± 0.50	27.00 ± 0.50
RH							
Low DCAD	68.00 ± 2.00	61.00 ± 1.00	55.00 ± 1.00	48.50 ± 3.50	45.00 ± 4.00	51.00 ± 1.00	70.00 ± 1.00
High DCAD-7	74.50 ± 2.50	57.50 ± 1.50	51.00 ± 2.00	48.00 ± 1.00	46.50 ± 0.50	50.00 ± 2.00	65.00 ± 4.00
High DCAD-18	70.00 ± 2.00	61.50 ± 1.50	48.50 ± 0.50	40.50 ± 1.50	45.00 ± 4.00	40.00 ± 2.00	65.00 ± 3.00
THI							
Low DCAD	76.01 ± 0.51	78.05 ± 0.84	78.59 ± 0.53	80.84 ± 0.70	81.50 ± 0.54	79.97 ± 1.16	77.74 ± 2.13
High DCAD-7	75.97 ± 1.27	77.57 ± 0.49	80.00 ± 1.63	81.17 ± 0.55	82.63 ± 0.37	81.77 ± 0.30	76.42 ± 1.21
High DCAD-18	75.52 ± 1.72	78.84 ± 1.63	80.23 ± 0.56	82.97 ± 0.25	81.94 ± 0.32	80.03 ± 0.25	76.42 ± 1.09

Ta=Ambient temperature, RH=Relative humidity, THI=Temperature humidity index. DCAD levels: Low DCAD=15.59 mEq/100 g DM; High DCAD-7 and high DCAD-18=89.05 mEq/100 g DM for day 7 and 18, respectively

### Data collection and measurements

**Figure-1:**
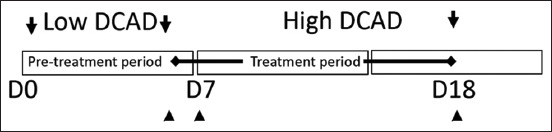
Experimental period uses for this experiment. Seven non-lactating goats were offered to low dietary cation and anion difference (DCAD) from day 1 to day 6 (arrows). On day 7, all animals were switched from low DCAD to high DCAD until the end of experiment (day 18; arrow). The measurement of meal pattern was conducted on day 6, 7, and day 18 (arrow heads).

Feeds offered and refusals were recorded every morning from day 1 to the end of the experiment. Eating patterns were continuously recorded for 24 h using a digital balance equipped with data processing software (PBA 665 and Weigh Term 231G, Mettler Toledo, Zürich, Switzerland). These digital balances were fixed under the goats’ feed containers. The actual weight of these containers was automatically checked and recorded by a personal computer every minute. The eating behavior pattern was recorded on day 6, day 7 (dietary switch), and day 18 of the experiment (Figure-1). Meals exceeding 5 g and separated by at least 15 min of non-feeding were defined as feed removals [9]. The recorded parameters included meal size, duration, frequency, and intermeal interval. Meal size was calculated at the beginning and end of meals, while meal duration refers to the time the animals spent eating in a day and was calculated as the average of all meal durations within 24 h. Intermeal interval denotes the interval between two successive meals and was calculated as the average of all intermeal intervals within a 24-h period. In addition, DMI was assessed every 2 h during the daytime (07.00–19.00) and nighttime and throughout day 18.

WI was measured daily throughout the experiment. Furthermore, WI and urine volume were measured every 2 h during the daytime (07.00–19.00) and nighttime and for 24 h on days 7 and 18. Subsequently, water balance was calculated by computing the difference between water input (comprising free water intake and water from the feed) and water output (urine volume), excluding water from metabolic processes and feces. Free water intake was calculated as the difference between the quantity of water offered and the amount refused.

Rectal temperature and respiration rate (RR) were measured on days 6, 7, and 17 at 07:00, 09:00, 11:00, 13:00, 15:00, 17:00, and 19:00. Rectal temperature was measured using a digital clinical thermometer (Digital Clinical Thermometer C202, Terumo, Tokyo, Japan), while RR was determined by counting flank movement within a 1-min timeframe**.**

Feed samples from both the feed offered and refusals were collected daily throughout the experiment and preserved at −20°C for subsequent analysis. At the end of the experiment, these samples were thawed and mixed thoroughly, with subsamples dried at 65°C until a constant weight was achieved. Crude protein and ash content were determined using the Association of Official Analytical Chemists method [11], while neutral detergent fiber and acid detergent fiber were determined following the methods recommended by a previous study by Van Soest *et al*. [12]. Sodium (Na^+^), potassium (K^+^), calcium (Ca^2+^), magnesium (Mg^2+^), chloride, and sulfate ion (SO_4_^2−^) content were determined following the procedures described by Nguyen *et al*. [8]. In summary, Na^+^, K^+^, Ca^2+^, and Mg^2+^ were analyzed using an atomic absorption spectrophotometer (Thermo iCE 3000 series, Cambridge, UK). Chloride was measured using colorimetric titration, and SO_4_^2−^ was determined using spectrophotometry (UV-VIS 1800 Shimadzu, Kyoto, Japan).

### Statistical analysis

All data are expressed as the mean ± standard error of the mean. The data for RR and Tr, as well as DMI, WI, and urinary excretion patterns, were analyzed using repeated two**-**way analysis of variance (ANOVA). The significance of the main effects, including RR, Tr, DMI, WI, and urinary excretion, was determined using the Tukey posttest. The data for the other parameters were analyzed using a one-way ANOVA, and significance was determined using the Tukey posttest. The significance level was set at p < 0.05. Data were analyzed using GraphPad Prism (GraphPad Software, Boston, MA, USA).

## Results

The experiment showed that the animals stayed under Ta and THI conditions from 13:00 to 17:00, with Ta ranging from 31.75 to 33.63 and THI ranging from 80.43 to 82.12, compared to other time points (Table-1).

**Figure-2:**
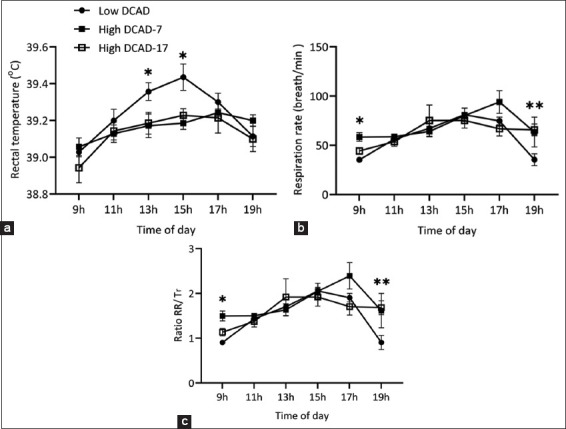
Effects of across dietary shift from low to high dietary cation and anion difference on (a) rectal temperature (Tr), (b) respiration rate (RR), and (c) ratio RR/Tr. *p < 0.05 and **p < 0.01.

Rectal temperature in the high DCAD group on days 7 and 17 was lower than that in the low DCAD group between 11:00 and 5:00 (Figure-2a, p < 0.05). Notably, during days 7 and 18, there was a significant increase in RR among goats fed with a high DCAD diet from 07:00 to 09:00, suggesting a potential physiological response to these specific dietary conditions (Figure-2b, p < 0.05). Similarly, a high DCAD diet also increased the RR/Tr ratio at 09:00 and 19:00 (Figure 2c, p < 0.05).

**Figure-3:**
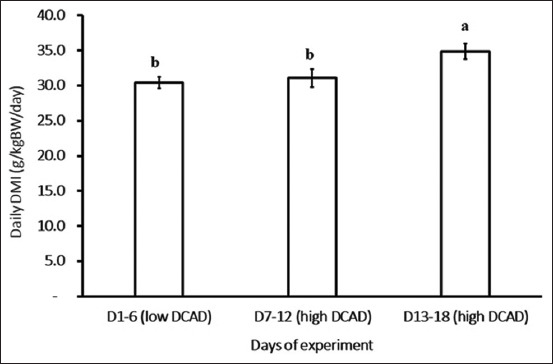
Effects of across dietary shift from low to high dietary cation and anion difference (DCAD) on daily dry matter intake (g**/**kg**/**BW). Goats fed low DCAD (15.59 mEq/100 g DM) from day 1 to day 6 and high DCAD (89.05 mEq/100 g DM) from day 7 to day 18.

We calculated the average daily DMI for the low DCAD group from days 0 to 6 and compared it to that of the high DCAD group from days 7 to 12 and from days 13 to 18. No significant difference in DMI was observed between the low and high DCAD groups from days 7 to 12 in this study. However, the DMI observed in the high DCAD group from days 12 to 18 was greater than that in the low and high DCAD groups from days 7 to 12 (Figure-3, p < 0**.**05).

DCAD supplementation affected meal patterns in this study. Increased meal size led to a higher total DMI in both groups on day 18, but this effect was not observed on day 7 (Table-3, p < 0.05). The increased daily meal size on day 18 resulted from the increased meal size during daytime, not nighttime. However, meal duration was unaffected by DCAD supplementation. Meal frequency and intermeal interval were similar across both groups for 24 h, during daytime and nighttime (Table-3, p > 0.05). The higher DMI during the daytime on day 18 was primarily due to increased intake from 07:00 to 09:00, although there was a slight reduction in intake from 15:00 to 17:00 (Table-4).

**Table-3:** Meal pattern after shift from low to high dietary cation and anion difference.

Items	Time	Low DCAD	High DCAD		p-value

			Day 7	Day 18	
DMI (g/kg BW/day)	24 h	30.17 ± 1.68^b^	32.14 ± 2.01^b^	39.71 ± 3.05^a^	0.02
	Day time	27.18 ± 1.55	30.36 ± 2.08	36.08 ± 3.52	0.065
	Night-time	3.00 ± 1.56	2.17 ± 0.37	3.87 ± 1.50	0.33
Meal size (g/kg BW)	24 h	1.56 ± 0.10^b^	1.85 ± 0.21^b^	2.50 ± 0.37^a^	0.05
	Day time	2.45 ± 0.10	3.14 ± 0.37	4.19 ± 0.74	0.06
	Night-time	0.69 ± 0.20	0.64 ± 0.13	0.86 ± 0.26	0.39
Meal duration (min)	24 h	26.86 ± 1.68	29.57 ± 2.31	27.29 ± 2.16	0.61
	Day time	33.29 ± 2.23	39.29 ± 3.69	37.14 ± 2.76	0.37
	Night-time	20.17 ± 2.33	20.86 ± 1.84	20.00 ± 1.41	0.87
Meal frequency	24 h	14.00 ± 0.90	12.86 ± 1.03	12.00 ± 1.23	0.43
	Day time	10.57 ± 0.43	9.86 ± 0.74	8.86 ± 0.67	0.18
	Night-time	3.17 ± 0.98	3.57 ± 0.37	3.29 ± 0.71	0.81
Inter meal interval (min)	24 h	87.29 ± 14.05	82.14 ± 21.39	97.57 ± 30.02	0.89
	Day time	27.00 ± 2.59	27.86 ± 2.76	32.43 ± 4.65	0.50
	Night-time	165.50 ± 27.24	86.71 ± 9.40	175.57 ± 53.01	0.18

Low DCAD=15.59 mEq/100 g DM; High DCAD=89.05 mEq/100 g DM; Dietary shift from low to high DCAD (15.59–89.05 mEq/100 g DM). DCAD=Dietary cation and anion difference, DMI=Dry matter intake, BW=Body weight. Means within the same row with different superscripts (a and b) differ significantly at p < 0.05

**Table-4:** Feed intake (g/kg BW) pattern at different time interval after shift from low to high DCAD.

Time	Low DCAD	High DCAD		p-value

		Day 7	Day 18	
07–09 h	7.58 ± 1.15^b^	8.54 ± 1.06^b^	12.33 ± 1.61^a^	0.044
09–11 h	3.81 ± 0.85	4.69 ± 0.50	4.37 ± 0.62	0.65
11–13 h	2.89 ± 0.35	2.44 ± 0.39	3.25 ± 0.37	0.33
13–15 h	6.15 ± 0.67	6.29 ± 0.91	9.07 ± 1.44	0.12
15–17 h	4.62 ± 0.60	4.61 ± 0.54	2.72 ± 0.68	0.06
17–19 h	2.46 ± 0.43	4.03 ± 1.15	4.36 ± 0.89	0.28
19–06 h	2.55 ± 1.35	1.50 ± 0.55	2.69 ± 1.11	0.69
Total DMI	30.06 ± 1.71^b^	32.11 ± 2.03^b^	38.79 ± 2.18^a^	0.02

Low DCAD=15.59 mEq/100 g DM; High DCAD=89.05 mEq/100 g DM; Dietary shift from low to high DCAD (15.59–89.05 mEq/100 g DM). DCAD=Dietary cation and anion difference, DMI=Dry matter intake, BW=Body weight. Means within the same row with different superscripts (a and b) differ significantly at p *<* 0.05

No impact of DCAD supplementation was observed on daily WI throughout the experiment. However, distinct differences in drinking patterns emerged between the low DCAD and high DCAD groups during the dietary shift on day 7. Animals fed with high DCAD diets from day 7 consumed more water between 07:00 and 09:00 (Table-5, p < 0.05). Conversely, animals fed with high DCAD on day 7 showed lower WI during nighttime compared to low DCAD and high DCAD on day 18 (Table-5, p < 0.05).

**Table-5:** Water intake (g/kg BW) pattern at different time interval after shift from low to high DCAD.

Time	Low DCAD	High DCAD		p-value

		Day 7	Day 18	
07–09 h	26.02 ± 2.92	40.04 ± 5.68	28.99 ± 3.06	0.06
09–11 h	10.43 ± 2.77	17.87 ± 3.70	11.19 ± 2.53	0.19
11–13 h	11.16 ± 1.95	9.92 ± 1.92	15.91 ± 4.82	0.40
13–15 h	21.49 ± 3.93	25.34 ± 7.68	16.42 ± 3.87	0.52
15–17 h	18.12 ± 7.06	13.97 ± 3.22	15.38 ± 2.77	0.82
17–19 h	8.04 ± 3.64	6.80 ± 3.20	5.02 ± 2.66	0.80
19–06 h	5.30 ± 2.65^a^	0.55 ± 0.23^b^	8.41 ± 2.38^a^	0.05
Total WI	100.55 ± 17.92	114.48 ± 10.05	101.08 ± 8.18	0.69

DCAD=Dietary cation and anion difference, WI=Water intake, BW=Body weight. Means within the same row with different superscripts (a and b) differ significantly at p < 0.05

Total daily urine volume and urinary excretion patterns at various time points remained unaffected by DCAD supplementation after transitioning from low to high DCAD diets (Table-6, p > 0.05). However, water balance tended to increase (Table-7) on day 7 (high DCAD) compared to days 6 (low DCAD) and 18 (high DCAD). This difference primarily resulted from the morning water balance in this study (Table-7, p < 0.05).

**Table-6:** Urinary excretion (g/kg BW) pattern at different time interval after shift from low to high DCAD.

Time	Low DCAD	High DCAD		p-value

		Day 7	Day 18	
07–09 h	2.32 ± 0.57	3.06 ± 1.00	4.56 ± 0.68	0.14
09–11 h	4.50 ± 1.57	4.15 ± 0.66	2.90 ± 1.25	0.63
11–13 h	4.86 ± 1.95	4.72 ± 2.13	6.51 ± 1.01	0.73
13–15 h	3.63 ± 1.99	5.02 ± 2.46	5.23 ± 1.67	0.84
15–17 h	9.82 ± 3.71	6.49 ± 1.43	5.19 ± 0.68	0.37
17–19 h	8.57 ± 2.39	6.06 ± 1.13	3.63 ± 0.51	0.11
19–06 h	33.19 ± 3.48	39.02 ± 8.72	49.88 ± 7.48	0.25
Daily Uex.	68.32 ± 18.94	68.51 ± 10.26	77.91 ± 9.35	0.85

Low DCAD=15.59 mEq/100 g DM; High DCAD=89.05 mEq/100 g DM; Dietary shift from low to high DCAD (15.59–89.05 mEq/100 g DM). DCAD=Dietary cation and anion difference, BW=Body weight

**Table-7:** Effects of across dietary shift from low to high DCAD on water balance.

Items	Time	Low DCAD	High DCAD		p-value

			Day 7	Day 18	
Water input (g/kg BW)	Morning	73.97 ± 5.90	96.76 ± 8.22	92.92 ± 5.55	0.058
	Afternoon	72.05 ± 11.63	73.68 ± 7.85	67.05 ± 10.35	0.89
	Night-time	10.01 ± 3.22	3.32 ± 0.94	13.37 ± 3.90	0.08
	24 h	156.04 ± 18.14	173.76 ± 11.00	173.09 ± 10.45	0.59
Water output (g/kg BW)	Morning	11.68 ± 2.41	11.93 ± 2.50	13.97 ± 1.45	0.72
	Afternoon	22.02 ± 6.82	17.57 ± 3.01	14.06 ± 1.91	0.46
	Night-time	33.19 ± 3.48	39.02 ± 8.72	49.88 ± 7.48	0.25
	24 h	68.32 ± 18.94	68.51 ± 10.26	77.91 ± 9.35	0.85
Water balance (g/kg BW)	Morning	62.29 ± 4.04^b^	84.83 ± 7.52^a^	78.95 ± 5.11^b^	0.03
	Afternoon	50.03 ± 8.41	56.11 ± 9.17	52.99 ± 10.20	0.90
	Night-time	−23.17 ± 4.33	−35.70 ± 8.41	−36.51 ± 5.04	0.58
	24 h	87.72 ± 13.07	105.24 ± 9.64	95.18 ± 12.27	0.26

DCAD=Dietary cation and anion difference, BW=Body weight. Means within the same row with different superscripts (a and b) differ significantly at p < 0.05

## Discussion

The observed eating, drinking, and urinary patterns suggested that high DCAD supplementation can mitigate the effect of HTa conditions in non-lactating goats. Unlike in lactating goats, supplementation with high DCAD during the low metabolic rate period in non-lactating goats mitigated the effects of HTa and helped to maintain their body temperature. This effect seemingly stems from the positive body water balance, in which the high DCAD group can utilize for evaporative heat dissipation through panting.

In this study, the goats were kept under a THI of 82.12 at 15.00, showing a higher RR and Tr at this time than at other time points in the day. However, the animals seemed comfortable in the early morning and late afternoon due to the low THI [10]. Consequently, RR and Tr across all groups increased throughout the day in response to Ta and THI (Figures-2a and b). Notably, goats fed a high DCAD diet had lower Tr s at 13:00 and 15:00 compared to those on a low DCAD diet. The lower Tr in the high DCAD group might be attributed to increased water balance in the morning (07:00–13:00), contrary to the low DCAD group in this study. This finding was similar to previous study on lactating goats [10], although animals in this study had a lower metabolic rate than those in the previous studies (non-lactating and non-pregnant vs. lactating). In addition, our study revealed that RR was greater in the high DCAD groups compared to the low DCAD groups during the cooler parts of the day (07:00 and 19:00). This finding aligned with our results in the previous study that lactating goats supplemented with high DCAD showed increased RR compared to the low DCAD group [10]. Changes in RR due to the influence of environmental temperature may not align with changes in body temperature. Moreover, RR in animals is influenced by the levels of carbon dioxide in the blood, and high pCO_2_ levels can stimulate an increase in the rate and depth of respiration [13]. These findings align with the previous studies by West *et al*. [5] and Do Nguyen *et al*. [14] on dairy cows which showed that supplementation with high DCAD (from 120.4 to 456 mEq/kg DM) increased pCO_2_ and RR.

We observed that animals in the high DCAD group consumed more feed from days 15 to 18. This increase in DMI may be due to improved ruminal function and enhanced nutrition digestibility associated with a high DCAD intake [9, 15]. Similar results were observed in the previous studies by West *et al*. [5] and Nguyen *et al*. [9] involving dairy cows and goats. In addition, Delaquis and Block [6] observed that DMI increased when dairy cattle were fed diets with DCAD levels ranging from 5.55 to 25.81 mEq/100 g of DM during early lactation or from 14.02 to 37.27 mEq/100 g of DM during mid lactation. However, these effects did not occur during late lactation. However, González *et al*. [16] reported that increasing DCAD levels, particularly NaHCO_3_ supplementation up to 5% of the diet, did not significantly affect total DMI. As reported in the previous studies by Zimpel *et al*. [17] and Lopera *et al*. [18], decreased DMI was observed when non-lactating pregnant cows were fed a negative DCAD diet. Our study results show that higher daytime meal sizes contributed to an increase in DMI on day 18, which contrasts with the previous research where increased meal sizes were observed in both daytime and nighttime feeding. For instance, González *et al*. [19] found that beef supplemented with high DCAD resulted in increased meal size and decreased meal frequency, ultimately leading to unchanged total DMI. However, in this study, goats fed high DCAD on day 18 exhibited increased meal size while maintaining an unchanged meal frequency, contributing to an increase in total DMI. In principle, animals fed with high DCAD have increased Na^+^ and K^+^ intake, which may increase ruminal osmolality in this study. This may be confirmed by the high DCAD group eating less from 15.00 to 17.00 on day 18 than the low DCAD group (Table-4, p = 0.06).

In addition, our study showed that high DCAD levels impacted eating behavior (DMI and meal patterns) from days 15 to 18 (the 2^nd^ week after the dietary shift from low to high DCAD) but not on day 7 (dietary shift). Treesukosol and Moran [20] found that rats increased their feed intake and meal patterns within the 1^st^ week after a dietary shift from a low-fat to a high-carbohydrate diet, exhibiting different eating behaviors from the animals in the current experiment. However, it’s important to note that our previous study didn’t determine whether pre-absorptive or post-absorptive signals were the crucial mechanisms behind the effect of high DCAD on eating patterns [21, 22]. The high impact of DCAD on eating patterns might stem from signals such as satiation hormones and plasma metabolites after eating rather than factors related to palatability. This is because the basic taste perception effect of pre-absorptive signals can manifest in the early phase of the dietary shift [20], as was observed in this study from days 7 to 18 with the provision of a high DCAD diet. A significant increase in DMI and meal patterns was observed from days 15 to 18 (the 2^nd^ week after the dietary shift from low to high DCAD) but not on day 7. It is crucial to highlight that a high DCAD diet has been shown to result in an augmented meal size in goats. This observation implies that high DCAD levels are advantageous for goats, thereby endorsing the notion that integrating this formulation into routine management practices, particularly during the summer, can contribute positively to livestock welfare.

Interestingly, no significant effect of high DCAD on daily WI was observed in this study. While some studies have reported that high DCAD did not impact WI due to an unchanged DMI [16], other experiments have found that high DCAD increased both WI and DMI in high-temperature environments [9, 23]. Our study results show that goats in the high-DCAD-7 group tended to increase WI from 07:00 to 09:00 h and reduced WI during the nighttime compared to the low DCAD group. This may be due to higher Na^+^ and K^+^ intake from the high-DCAD-7 group. In addition, our study showed that goats from the high-DCAD-18 group consumed more water from feed (data not shown) than from daily WI or the 2-h drinking water measurements. This suggests that goats might regulate ruminal osmolality through increased consumption of drinking water [24] or water from feed.

The total urine volume and urinary excretion patterns were similar between the low and high DCAD groups in our study. This may be attributed to the goats’ inclination to conserve water and regulate Tr during the hottest parts of the day. Throughout this experiment, the total water input and output patterns remained consistent among treatments, resulting in a consistent daily water balance between the low and high DCAD groups. However, the morning water input (07:00–13:00 h) tended to be higher in the high DCAD group than in the low DCAD group, although water output remained similar among the treatments. Therefore, the morning water balance was greater in the high DCAD group compared to the low DCAD group.

Conversely, the afternoon and nighttime water input and output were unaffected by high DCAD supplementation. Consequently, the water balance during these periods was similar among the treatments in our study. The increased water balance was beneficial in moderating the rise in body temperature in dairy goats under HTa conditions [10]. Our study found that Tr in the high DCAD group between 13:00 and 15:00 was significantly lower than that in the low DCAD group (p < 0.05, Figure-2a), corroborating similar findings in the previous study on dairy cows [25]. However, despite the higher water intake and urine volume observed in those experiments, other studies have reported no effect of high DCAD on the water balance in both dry and lactating dairy cows [7]. Our experiment indicated that goats fed high DCAD diets demonstrated improved water balance, contributing to the maintenance of normal body temperature during the hottest parts of the day. Note that our current experiment aimed to investigate the effect of DCAD formulation during the transition period from low to high levels in non-lactating goats. Given the short duration of this experiment, measuring the body water compartments, which is a major mechanism for evaporative heat dissipation, was challenging [10]. Therefore, our interpretation of the effect of a high DCAD formulation on Tr through increased respiratory heat dissipation needs confirmation, along with information regarding the impact of high DCAD on body water distribution and kinetics. This includes the movement of reserved water from transcellular spaces (such as the rumen [10]) to the intravascular space during a gradual increase in Ta.

## Conclusion

Our study results indicate that non-lactating and non-pregnant goats undergoing a transition from low to high DCAD levels under tropical conditions experienced improved morning water balance. The findings also showed that a high DCAD formulation contributes to an increase in meal size, supporting the idea that a high DCAD diet can enhance the welfare of goats under tropical farming conditions. These findings are beneficial in aiding the animals to adapt to HTa by potentially maintaining a lower Tr within the high DCAD group. In addition, reducing the negative effects of HTa helps the goats consume more feed, as observed in this study.

## Authors’ Contributions

TN, NC, SC, and ST: Conception and design of the study. NC, SC, and ST: Reagents/materials/analysis tools. TN and SS: Performed animal experiments. TN and ST: Statistical analysis and wrote and revised the manuscript. All authors have read, reviewed, and approved the final manuscript
